# PACAP38 and PAC_1_ receptor blockade: a new target for headache?

**DOI:** 10.1186/s10194-018-0893-8

**Published:** 2018-08-07

**Authors:** Eloisa Rubio-Beltrán, Edvige Correnti, Marie Deen, Katharina Kamm, Tim Kelderman, Laura Papetti, Simone Vigneri, Antoinette MaassenVanDenBrink, Lars Edvinsson, On behalf of the European Headache Federation School of Advanced Studies (EHF-SAS)

**Affiliations:** 1 Division of Vascular Medicine and Pharmacology, Department of Internal Medicine, Erasmus University Medical Center, Rotterdam, The Netherlands; 2 Department of Child Neuropsychiatry, University of Palermo, Palermo, Italy; 3 Danish Headache Center, Department of Neurology, Rigshospitalet Glostrup, Glostrup, Denmark; 4 Department of Neurology, University Hospital, LMU Munich, Munich, Germany; 5 Department of Neurology, Ghent University Hospital, Ghent, Belgium; 6 Headache Center, Bambino Gesù Children’s Hospital, IRCCS, Rome, Italy; 7 Department of Experimental Biomedicine and Clinical Neurosciences, University of Palermo; Pain Medicine Unit, Santa Maria Maddalena Hospital, Occhiobello, Italy; 8 Department of Internal Medicine, Institute of Clinical Sciences, Lund University, Lund, Sweden

**Keywords:** PACAP, PAC_1_ receptor, Migraine, Prophylactic treatment

## Abstract

Pituitary adenylate cyclase activating polypeptide-38 (PACAP38) is a widely distributed neuropeptide involved in neuroprotection, neurodevelopment, nociception and inflammation. Moreover, PACAP38 is a potent inducer of migraine-like attacks, but the mechanism behind this has not been fully elucidated.

Migraine is a neurovascular disorder, recognized as the second most disabling disease. Nevertheless, the antibodies targeting calcitonin gene-related peptide (CGRP) or its receptor are the only prophylactic treatment developed specifically for migraine. These antibodies have displayed positive results in clinical trials, but are not effective for all patients; therefore, new pharmacological targets need to be identified.

Due to the ability of PACAP38 to induce migraine-like attacks, its location in structures previously associated with migraine pathophysiology and the 100-fold selectivity for the PAC_1_ receptor when compared to VIP, new attention has been drawn to this pathway and its potential role as a novel target for migraine treatment. In accordance with this, antibodies against PACAP38 (ALD 1910) and PAC_1_ receptor (AMG 301) are being developed, with AMG 301 already in Phase II clinical trials. No results have been published so far, but in preclinical studies, AMG 301 has shown responses comparable to those observed with triptans. If these antibodies prove to be effective for the treatment of migraine, several considerations should be addressed, for instance, the potential side effects of long-term blockade of the PACAP (receptor) pathway. Moreover, it is important to investigate whether these antibodies will indeed represent a therapeutic advantage for the patients that do not respond the CGRP (receptor)-antibodies.

In conclusion, the data presented in this review indicate that PACAP38 and PAC_1_ receptor blockade are promising antimigraine therapies, but results from clinical trials are needed in order to confirm their efficacy and side effect profile.

## Review

### Discovery of PACAP

The description of the pituitary adenylate cyclase activating polypeptide-38 (PACAP38) was made by Arimura and his team in 1989, following the extraction of the peptide from more than 4000 samples of ovine hypothalamus. After the isolation, its characterization showed that it was formed by 38 amino acids, with a 68% homology with vasoactive intestinal peptide (VIP), described almost twenty years earlier [1]. Subsequently, the peptide was synthesized and shown to activate adenylyl cyclase (AC) in cultures of rat pituitary cells, thereby obtaining its name as pituitary adenylate cyclase activating polypeptide. A year later, a fragment of PACAP38 with similar AC activation profile was isolated. This was formed by 27 amino acids and thus named PACAP27 [2]. That same year, cloning of cDNA from ovine PACAP38 revealed that the amino acid sequence of the mature human PACAP38 was identical to that of the ovine. In addition, later studies showed that it was identical in all mammals [3], suggesting that it has been conserved during evolution.

This review will give an overview of PACAP, its complex signaling pathway, the role PACAP and its receptors have in physiological conditions and their involvement in some disorders, with special focus on migraine. Moreover, the preclinical results of PACAP (receptor) blockade in migraine models, the side effects that could be expected in clinical trials, and the considerations that must be taken if PACAP (receptor)-antibodies are effective for migraine treatment will be discussed.

### Pharmacology

PACAP belongs to a wider group of peptides called the VIP/glucagon/growth hormone releasing factor/secretin superfamily. The ADCYAP1gene, located on chromosome 18, encodes PACAP; initially, a proprotein is expressed, and later processed to form a 38 amino acid peptide (PACAP38) with a cleavage-amidation site that can generate a 27-residue-amidated fragment (PACAP27). In mammals, the most prevalent form is PACAP38 [4], therefore, in this review PACAP38 will be referred as PACAP unless stated otherwise.

**Fig. 1:**
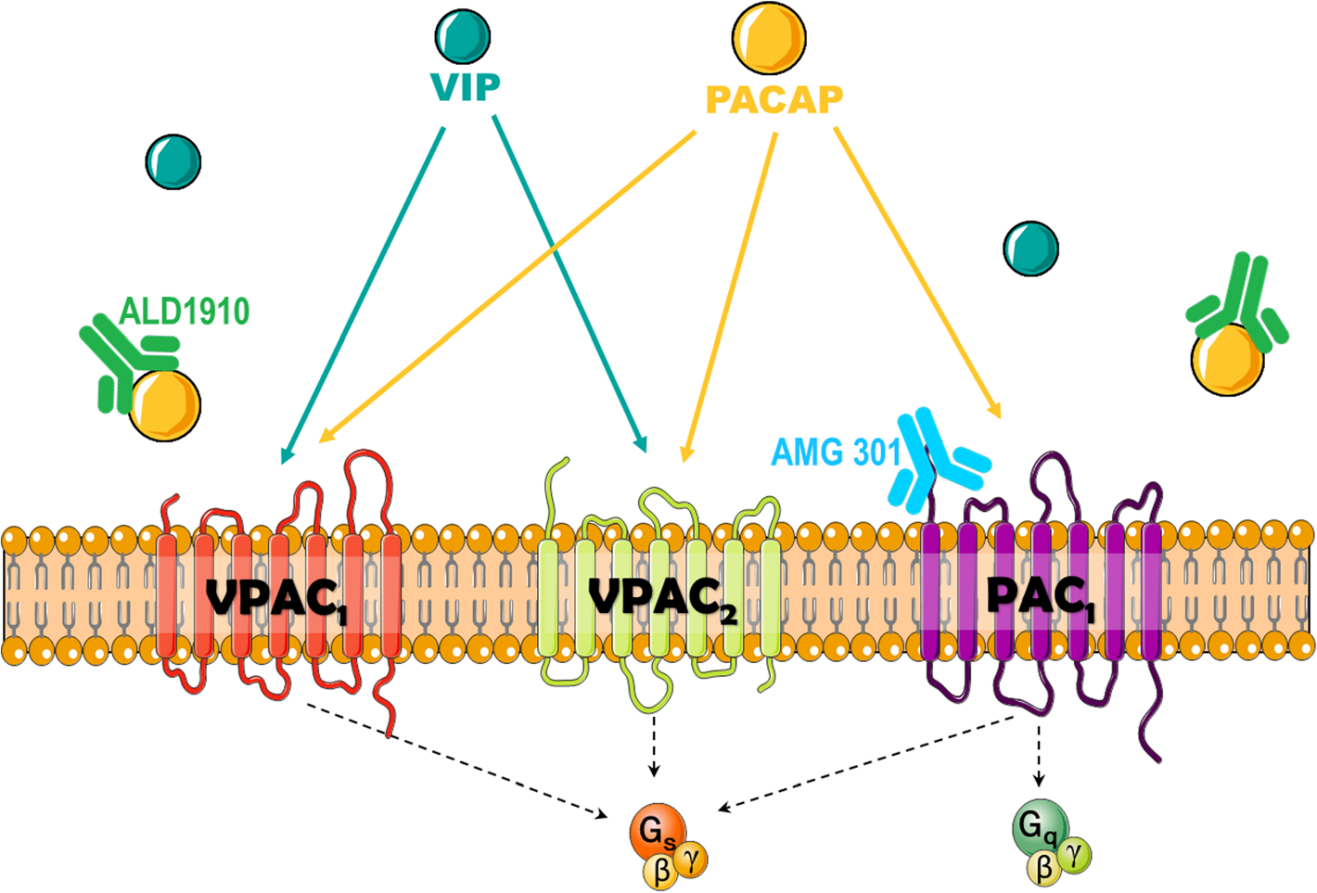
PACAP receptors. Three receptors to PACAP have been described: VPAC_1_, VPAC_2_ and PAC_1_. VIP and PACAP show similar affinity for VPAC_1_ and VPAC_2_, whereas PACAP is 100-fold more selective for PAC_1_ receptor. The antibodies developed for prophylactic antimigraine treatment bind either to PACAP (PACAP38, ALD1910) or to the PAC_1_ receptor (AMG 301)

Three PACAP receptors have been described: VPAC_1_, VPAC_2_ and PAC_1_, all coupled to G-proteins (Fig. 1). VPAC_1_ and VPAC_2_ receptors present equal affinity for PACAP and VIP and their activation stimulates AC. On the other hand, PAC_1_ receptor is 100 times more selective for PACAP and presents a complex signaling pathway [4].

Alternative splicing of the PAC_1_ receptor gene results in several isoforms. These receptor variants are characterized by shorter extracellular domains (PAC_1_*short*, PAC_1_*veryshort*), different inserts in an intracellular loop important for G-protein interaction (PAC_1_*null*, PAC_1_*hip*, PAC_1_*hop1*, PAC_1_*hop2*, PAC_1_*hiphop1*, PAC_1_*hiphop2*) and/or discrete sequences located in transmembrane domains II and IV (PAC_1_*TM4*) [5–8]. Of relevance, in humans, twelve homologues have been reported [7, 9–11], which have been reviewed elsewhere [12, 13]. For each splice variant, PACAP38 and PACAP27 present similar affinity and potency for AC and phospholipase C (PLC) stimulation, but different efficacy (i.e. maximal effect) of PLC responses [14, 15]. Although in several processes the activation of AC or PLC can result in similar “stimulatory” responses, in smooth muscle cells (e.g. blood vessels), activation of AC leads to vasodilation, whereas PLC activation results in vasoconstriction. This plays an important role in disorders such as migraine, where expression of a PAC_1_ receptor isoform with a lower PLC efficacy could favor AC stimulation, thus facilitating vasodilatory responses in cranial blood vessels [16, 17].

To study PAC_1_ receptor-mediated responses, selective agonists and antagonists are used. Currently, one selective agonist has been described, maxadilan [18, 19] and three antagonists M65, Max.d.4 and PACAP6–38 [20]. However, no study has investigated whether such compounds are selective for one PAC_1_ receptor variant, or whether they bind to all isoforms. Moreover, PACAP6–38 also binds to the VPAC_2_ receptor, and, together with M65, has been shown to behave as agonist of the PAC_1_ receptor in certain tissues [21, 22]. Hence, novel selective pharmacological tools are needed to characterize PAC_1_ receptor-mediated responses. Indeed, an antibody against the PAC_1_ receptor, such as AMG 301, could be useful for characterization; however, it is yet not clear wheter this antibody is selective for one specific variant. If the antibody would be selective for one of the splice variants, this may affect its therapeutic potential, in particular if there are different splice variants expressed in different human populations. On the other hand, different splice variants might hypothetically offer the possibility of designing a drug that would selectively affect the PAC_1_ receptor in the trigeminovascular system, while not affecting PAC_1_ receptors at other sites in the body, thus reducing its potential side effects.

### Physiological roles of PACAP and the PAC_1_ receptor

Preclinical studies have shown that PACAP and PAC_1_ receptors are widely distributed, both centrally and peripherally. It is therefore not surprising that PACAP is described as a (neuro)hormone, neurotransmitter, neuromodulator, neurotrophic factor and immunomodulator [13]. As the PAC_1_ receptor is currently under investigation for migraine treatment, only the distribution of this receptor will be reviewed, while the distribution of VPAC_1/2_ receptors has been reviewed extensively elsewhere [13, 23, 24].

#### PACAP/PAC_1_ receptor in the central nervous system

PACAP fibers and PAC_1_ receptors are widely expressed throughout the central nervous system (CNS) with the highest density of both in the hypothalamus and supraoptic nucleus [25–31]. In accordance with this, PAC_1_ receptor activation has been associated with release of vasopressin and regulation of drinking behavior [32, 33], decrease of food intake [34–36], modulation of the sleep/wake cycle [37, 38], clock gene expression [38], melatonin synthesis stimulation [39], sexual maturation [40, 41], stress and sexual behavior [41, 42], learning [43], pain processing [44] and psychomotor responsiveness [45] .

Of special interest for migraine, both PACAP fibers and the PAC_1_ receptor are present in the paraventricular nucleus of the hypothalamus, the ventrolateral periaqueductal gray, the locus coeruleus, the solitary nucleus, the trigeminal nucleus caudalis (TNC) and the trigeminal ganglion (TG). These structures have all been associated with nociception and/or migraine pathophysiology [23, 46–49].

#### PACAP/PAC_1_ receptor in the periphery

Peripherally, PACAP fibers and/or cell bodies have been described in acrosome caps of primary spermatocytes, mature spermatids, in the testis, epithelial cells from epididymal tubules, the ovaries, mammary glands, in stromal stem cells and terminal placental villi, where the amount of PACAP mRNA increases with the progression of pregnancy [50–52]. Similarly, PAC_1_ receptors have been described in spermatids, the penile corpus cavernosum, the ovaries, the chorionic vessels and in stromal and decidual cells of the placenta [51, 53–55]. Considering the presence of PACAP and PAC_1_ receptors also in hypothalamus and pituitary, an important role in modulation of the hypothalamo-pituitary-gonadal axis is suggested.

PACAP fibers and cell bodies are also found in the adrenal gland, pancreas, epithelium and smooth muscle cells of the urinary tract, the bladder, urethra, larynx, lungs, gastrointestinal smooth muscle cells, duodenal mucosa, thymus, spleen and innervating vascular smooth muscle cells [23, 26, 56–67]. PAC_1_ receptors have been described in the adrenal medulla, pancreas, liver, lungs, enterochromaffin-like cells, thymus and vascular smooth muscle cells [47, 56, 62, 67–70].

Due to their vast distribution peripherally, PACAP and the PAC_1_ receptor are involved in a variety of physiological processes, such as regulation of adrenaline release [71], stimulation of adipocyte thermogenesis [72], lipid metabolism [73], metabolic stress adaptation [74], glucose and energy homeostasis [75], renin production [76, 77] and inflammatory responses [78]. Furthermore, PACAP and the PAC_1_ receptor have a crucial role in the long-term maintenance of neurogenic vasodilation in the periphery and in the homeostatic responses to cerebral, retinal, cardiac, hepatic, intestinal and renal ischemic events [79–88]. This topic has been extensively reviewed elsewhere [89].

### PACAP and PAC_1_ receptor in pathophysiological conditions

Besides being involved in several physiological processes, PACAP is thought to contribute to the pathophysiology of several conditions.

PACAP has been associated with regulation of inflammatory processes. In an arthritis model, PACAP^−/−^ mice showed absence of arthritic hyperalgesia and reduction of joint swelling, vascular leakage and inflammatory cell accumulation. In the late phase of the disease, immune cell function and bone neoformation were increased [90]. In rheumatoid arthritis, the vasodilatory effects of PACAP through activation of the PAC_1_ receptor facilitated plasma leakage, edema formation, and leukocyte migration [91, 92]. Furthermore, PACAP^−/−^ mice developed more severe inflammation and tumors in a model of colitis [78]. In preclinical models, upregulation of PACAP and its receptors in micturition pathways contributed to the development of urinary bladder dysfunction, including symptoms of increased voiding frequency and pelvic pain [58], suggesting a role in low urinary tract dysfunction. In the nervous system, studies demonstrated anxiogenic actions of PACAP and the possibility of blocking anxiety-related behaviors with PAC_1_ receptor antagonists [93–95]. In patients with post-traumatic stress disorder (PTSD), blood levels of PACAP correlated with severity of stress-related symptoms [96], and in females, a single nucleotide polymorphism in the estrogen response element of the PAC_1_ receptor gene is predictive of PTSD diagnosis [97].

Furthermore, PACAP plays a complex role in pain transmission. At the peripheral sensory nerve terminals, pro- and anti-nociceptive effects are observed; while in CNS, central sensitization, increase of neuronal excitation and induction of chronic pain have been described [98]_._ In an acute somatic and visceral inflammatory model, PACAP decreased pain transmission; however, after application in the spinal cord, a transient induction of analgesia was followed by long-lasting algesia [99]. Moreover, injection of PACAP into the paraventricular nucleus of hypothalamus increased the activity of the TNC, an effect which was inhibited by the PAC_1_ receptor antagonist [48]. Although it has been shown that PACAP is actively transported through the blood-brain barrier (BBB), it is rapidly degraded or returned by efflux pumps [100]. Thus, a direct central action of peripheral PACAP is unlikely.

Although the role of PACAP in pain processing remains elusive, clinical data strongly suggest the involvement of PACAP in the pathophysiology of migraine and cluster headache (CH) (see also [101, 102]). Recent evidence of a correlation between a genetic variant of the PAC_1_ receptor gene (ADCYAP1R1) and susceptibility to CH was demonstrated [103]. Another study identified a relationship between altered PACAP levels in peripheral blood and different types of headache [104]. Further, two studies reported low interictal plasma levels of PACAP in migraine and CH when compared to controls [105, 106]. Particularly, a detailed analysis of PACAP mRNA expression in peripheral blood mononuclear cells detected a significantly lower level of PACAP in migraine patients compared to healthy controls, with no significant differences revealed between the control group and tension-type headache, CH or medication overuse headache groups. Interestingly, PACAP increased ictally in jugular or cubital blood of migraine [105, 107, 108] and CH patients [93, 106], and levels decreased as headache ameliorated after sumatriptan administration [108]. Finally, when administered to migraine patients, PACAP induced an instant headache in 90% of patients, which was later followed by a delayed headache similar to a migraine-like attack in two thirds of the subjects [109]. This has led to study the role of PACAP in migraine pathophysiology as will be discussed in the next section.

### PACAP in migraine pathophysiology

The use and development of experimental animal and human models of headache, migraine in particular, have provided invaluable insight into the pathophysiological mechanisms underlying headache disorders [110, 111]. To investigate the molecular mechanisms behind the headache-inducing effects of PACAP, a number of animal studies have been conducted. Additionally, several human studies have been performed, some of these in combination with imaging techniques. In the following sections, both human and animal studies investigating the headache-related effects of PACAP will be reviewed.

#### Human studies

The headache-inducing effect of PACAP was first reported in a study on cerebral blood flow in healthy volunteers, where 10 out of 12 participants reported mild to moderate headache after PACAP infusion [112]. A double-blind, randomized, placebo-controlled, crossover study later showed that 12 out of 12 healthy subjects and 11 out of 12 migraine patients reported headache after intravenous infusion of PACAP, compared to two and three, respectively, after placebo [109]. Further, two healthy subjects and one migraine patient reported a migraine-like attack within 1 h after infusion, whereas six migraine patients reported a migraine-like attack after a mean of 6 h (range 2–11 h) after infusion. This study also found dilation of middle cerebral artery (MCA) and the superficial temporal artery after PACAP infusion.

The role of vasodilation in PACAP-induced headache was further explored in a magnetic resonance angiography (MRA) study in healthy volunteers [113]. Eight out of nine participants reported an immediate headache and 100% reported a delayed headache after PACAP infusion. Further, over a 5 h period PACAP induced a sustained dilation of the *extracranial* middle meningeal artery (MMA) but no change in intracerebral MCA. Collectively, these studies support the notion that PACAP induces headache via sustained vasodilation. In another MRA study, PACAP infusion induced headache in 91% of included migraine patients, and 73% reported migraine-like attacks compared to 82% and 18%, respectively, after VIP administration. Further, PACAP induced a long-lasting (> 2 h) dilation of extracranial arteries, whereas the dilation caused by VIP normalized after 2 h. In both cases, dilation of intracranial arteries was not observed. This further underlines prolonged extracranial vasodilation as the migraine inducing mechanism of PACAP [114]. Interestingly, in an in vitro study neither PACAP nor VIP were potent in inducing vasodilation of the intracranial portion of the human MMA [115].

In a resting-state magnetic resonance study, infusion of PACAP affected connectivity in the salience, the default mode and the sensorimotor network during migraine attacks. VIP had no effect on these networks [116]. Another study in migraine patients reproduced the induction of migraine-like attacks in 72% of patients and showed that PACAP induced premonitory symptoms in 48% of patients compared to 9% after CGRP [117], suggesting an effect on central PAC_1_ receptors. However, as described above, PACAP is rapidly degraded or transported back after actively crossing the BBB [100]; therefore, the premonitory symptoms could be mediated via activation of a central structure that is not protected by the BBB.

Two studies in migraine patients have further analysed plasma levels of markers of peptide release from parasympathetic (VIP) and sensory (CGRP) perivascular nerve fibres; mast cell degranulation (tumour necrosis factor alpha and tryptase); neuronal damage, glial cell activation or leakage of the BBB (S100 calcium binding protein B and neuron-specific enolase); and hypothalamic activation (prolactin, thyroid-stimulating hormone, follicle-stimulating hormone, luteinizing hormone and adrenocorticotropic hormone) after PACAP infusion [114, 118]. Only levels of VIP, S100 calcium binding protein B, prolactin and the thyroid-stimulating hormone were modified and did not differ between patients who developed migraine-like attacks and those who did not. However, it is important to consider that samples were obtained from the antecubital vein and it is not known yet if peripheral plasma changes reliably reflect cranial release of mediators.

The human studies point out PACAP as a key player in migraine pathophysiology [102]. As VIP does not induce migraine-like attacks, it is assumed that PACAP’s actions are mediated by PAC_1_ receptor activation. Nevertheless, it is still too early to rule out VPAC_1/2_ receptors as additional potential antimigraine targets, since no studies in humans have been performed with antagonists. Further, the short plasma half-life of VIP, two minutes (as compared to 6–10 min of PACAP [119]), could be the cause of its lack of migraine-inducing effects.

#### Animal studies

To characterize the exact receptor involved in PACAP-mediated actions, the vasodilatory effect of PACAP was elucidated in animal studies, showing that VIP, PACAP38 and PACAP27 induce vasodilation of the rat MMA in vivo [120, 121]. Interestingly, this effect was blocked by VPAC_1_ antagonists in the former [120] and VPAC_2_ antagonists in the latter [121]. Both studies found no effect of PAC_1_ antagonists on vasodilation. Similarly, in an in vitro study, PACAP induced vasodilation of the *human* middle meningeal and distal coronary arteries, and this effect was not modified by PACAP6–38 [115]. In contrast, an ex vivo study found that PAC_1_ antagonists reversed the PACAP-induced vasodilation in the rat MMA [17]. As mentioned previously, PAC_1_ receptor antagonists have shown agonistic behavior and affinity for VPAC_2_ receptors. This could explain the contradictory results observed in the MMA vasodilation studies. Therefore, different methods must be used to elucidate the receptors involved in migraine pathophysiology. For example, in a in vivo model of chronic migraine, induced by recurrent chemical dural stimulation, PAC_1_ receptor mRNA was shown to be increased in the TG, but not in the TNC, and no significant differences were found in the expression of the VPAC_1_ and VPAC_2_ receptors [122]. Moreover, in an in vivo rat model, intravenous administration of AMG 301, the PAC_1_ receptor antibody, inhibited evoked nociceptive activity in the trigemino-cervical complex, and the results were comparable to the inhibition observed with sumatriptan [123].

In addition to sustained vasodilation, mast cell degranulation has also been suggested as one of the headache-inducing mechanisms of PACAP. This hypothesis is based on findings from animal studies showing that PACAP degranulates mast cells from the rat dura mater [124]. Further, PACAP-induced delayed vasodilation of the rat MMA is attenuated in mast cell depleted rats [125]. Interestingly administration of VIP did not result in mast cell release of histamine from the dura [126]. However, as mentioned previously, no changes in peripheral blood markers of mast cell degranulation have been observed in migraine patients [114, 118].

Collectively, the animal studies confirm that PACAP induces vasodilation and suggest that this effect might be mediated through degranulation of mast cells. Also, recent results show that these effects are most likely exerted through activation of the PAC_1_ receptor. Due to the contradictory results, further studies are warranted to confirm this.

### PACAP (receptor) blockade as a therapeutic target

As shown above, PACAP seems to play an important role in migraine pathophysiology. Although the exact receptor involved has not yet been elucidated, some studies indicate that the PAC_1_ receptor is the most important [17, 48, 113, 117, 122, 123]. Therefore, both PACAP and PAC_1_ receptor have been suggested as novel targets for migraine treatment and possibly a new therapeutic option for patients who do not respond to CGRP (receptor) blocking drugs. Although both neuropeptides co-localize in the trigeminal ganglion [49], and could share some biological cascades, the PACAP-induced migraine attacks indicate an independent role of PACAP in the genesis of migraine.

In this light, the interest from pharmaceutical companies for blocking the PACAP/PAC_1_ receptor pathway has increased. There are two therapeutic approaches to inhibit PACAP: (i) PAC_1_ receptor antagonists or antibodies directed against this receptor; or (ii) antibodies directed against the peptide PACAP [102]. Since PAC_1_ receptor antagonists have been reported to act as agonists depending on the tissue (see Pharmacology), the antibodies seem a better option for blocking this receptor.

Currently, a phase 2a, randomized, double blind, placebo-controlled study is underway to evaluate the efficacy and safety of a PAC_1_ receptor antibody (AMG 301) in subjects with chronic or episodic migraine (Clinical trials identifier: NCT03238781, [127]). Unfortunately, no preliminary results have been published so far. Preclinical studies are also evaluating a monoclonal antibody (ALD1910) targeting PACAP38 for its potential in the treatment of migraine patients who have an inadequate response to therapeutics directed at CGRP or its receptor [128].

#### Potential side effects of PACAP/PAC_1_ receptor blockade

**Fig. 2:**
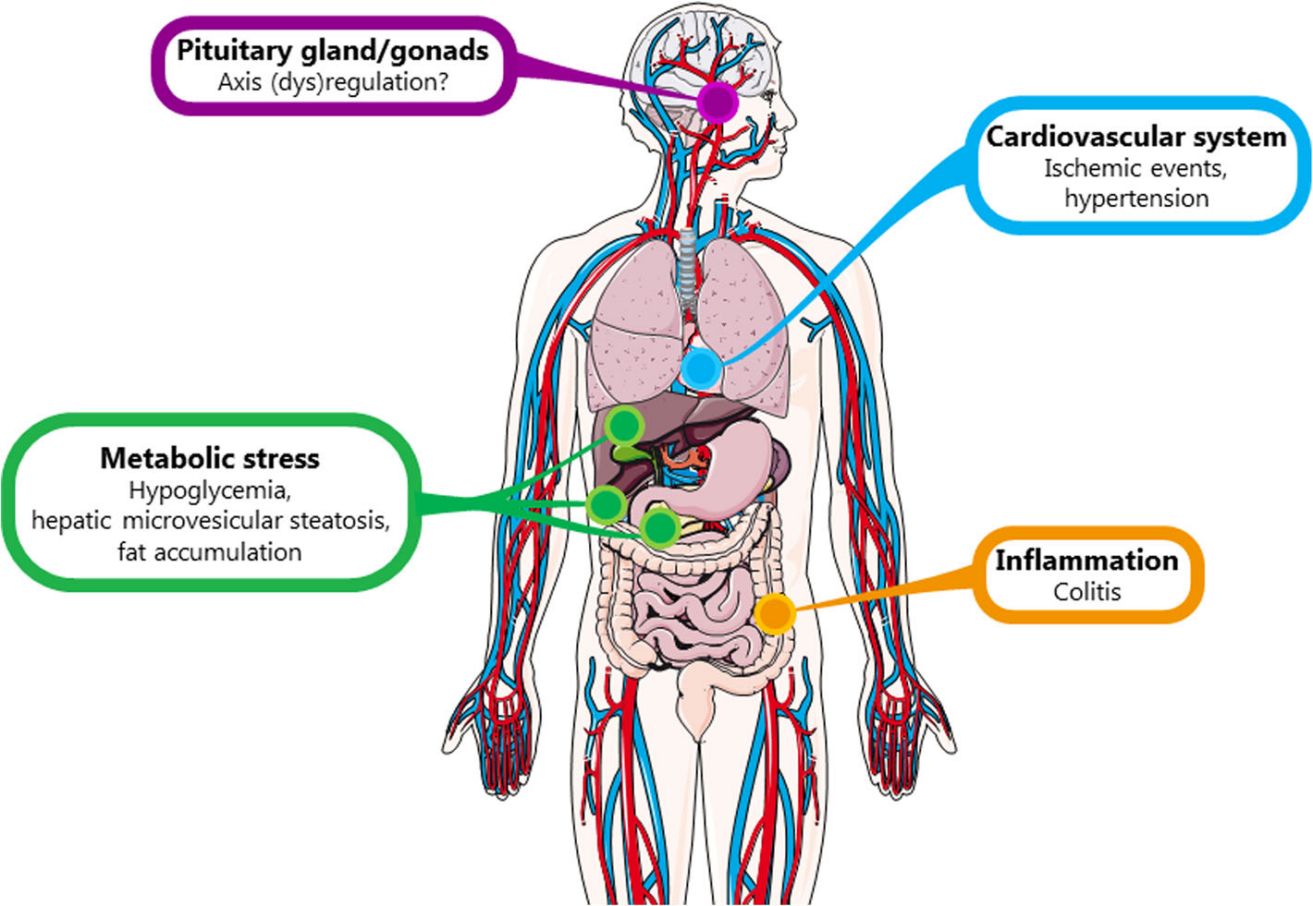
Possible side effects after long-term exposure to PACAP (receptor)-antibodies. An overview of the organ systems where PACAP and PAC_1_ receptor are present and the possible side effects that could be observed

Indeed, the possibility of a new therapeutic target for prophylactic migraine treatment is exciting; however, it is important to consider that PACAP and PAC_1_ receptor participate in numerous physiological processes (see Fig. 2). As antibodies are not likely to cross the BBB, only the possible side effects regarding peripheral blockade of PACAP and PAC_1_ receptor will be discussed.

As PACAP and PAC_1_ receptor are expressed throughout the components of the hypothalamo-pituitary-gonadal axis [50–52], and the pituitary gland is not protected by the BBB, a dysregulation of the functions of this axis could be a concern. Also, the immune system has been described to be regulated by activation of PAC_1_ receptor [61]. This, together with its participation in the modulation of inflammatory processes, could result in alterations in the immune response and increased production of pro-inflammatory cytokines [78, 129]. In accordance with this, in a mouse model of colitis, PACAP-deficient mice developed a more severe disease [78].

Blocking PACAP might also alter the response to metabolic stress. Studies with PACAP-deficient mice have shown a more profound and longer lasting insulin-induced hypoglycemia and a reduction in glucose-stimulated insulin secretion [74, 75]. Moreover, PACAP-deficient mice had hepatic microvesicular steatosis, intracellular fat accumulation in muscle and skeletal muscle and depletion of subcutaneous white fat [73].

Furthermore, PACAP and the PAC_1_ receptor participate in vasodilatory responses, renin release and regulation of cardiovascular function [77, 115, 125]. Although the density of VPAC_1/2_ and PAC_1_ receptors in coronary artery is less than that in cranial MMA [115], arguing for a limited role in cardiac ischemia, a protective role in ischemic events has been described. Thus, considering the increased cardiovascular risk that migraine patients present [130–133], careful monitoring of patients with preexisting cardiovascular risk factors is advised. However, similar concerns have been raised with the CGRP (receptor)-antibodies [134, 135], with no cardiovascular adverse events reported in the clinical trials [136].

### Further considerations

If the antibodies against the PAC_1_ receptor prove to be effective for the prophylactic treatment of migraine, some concerns should be addressed. Firstly, as previously discussed, it is important to consider the possible side effects of long-term blockade of PACAP/PAC_1_ receptor, with emphasis on the cardiovascular system, as migraine patients present a higher cardiovascular risk. Therefore, safety studies in patients with cardiovascular disease are needed. Moreover, the administration route of the antibody against the PAC_1_ receptor is subcutaneous, thus erythema, pruritus and mild pain in the injection site could be expected, as it has been observed with the CGRP (receptor) – antibodies [136]. Nevertheless, the monthly administration represents an advantage for treatment adherence.

It will also be important to define whether PAC_1_ receptor antibodies will really represent a therapeutic advantage for the patients that are not responding to the CGRP (receptor)-antibodies. Since studies have shown that PACAP and CGRP co-localize in structures relevant for migraine pathophysiology (e.g. trigeminal ganglion) [49], PACAP blockade may only be effective for the same patients to whom CGRP blockade is already effective. If a distinction can be made between patient groups this would also shed light on the pathophysiology of migraine, as it could distinguish between CGRP-associated or PACAP-associated migraine patients. Moreover, the PAC_1_ receptor sequence that is recognized by the antibody has not been disclosed, thus, the variants of the receptor to which the antibody binds are not known. If revealed, it would be interesting to study whether certain receptor isoforms predispose patients to present migraine, or whether the treatment will only be effective in patients with those isoforms.

Finally, as mentioned previously, it is still too early to rule out VPAC_1/2_ receptors as therapeutic targets for migraine treatment. Therefore, ALD1910, the antibody against PACAP38, currently undergoing preclinical studies [128], broadens the therapeutic options for migraine treatment. However, further safety studies should be addressed, as blocking PACAP38 would inhibit the actions of three different receptors, increasing the possibilities of adverse side effects.

## Conclusion

The possible role of PACAP/PAC_1_ receptor blockade as migraine treatment has been reviewed. All three PACAP receptors have been described in TG, TNC and (dural) arteries, structures previously related to migraine pathophysiology [47, 49]. Indeed, infusion of PACAP is able to induce migraine-like attacks [109]. Moreover, interictally, low plasma levels of PACAP have been described [105], while during a migraine attack, PACAP increases in jugular and cubital blood [105, 108] and decreases as headache ameliorates after sumatriptan administration [108].

Clinical studies have shown that infusion of VIP does not induce migraine-like headaches [114], therefore, it is considered that the possible receptor involved in PACAP actions is PAC_1_ receptor, as VIP has affinity for VPAC_1_ and VPAC_2_ receptors; although this could be attributed to pharmacokinetic (i.e. half-life), rather than pharmacodynamic aspects. Pharmacological characterization in preclinical studies has provided contradictory results, indicating a complex pharmacology of the PAC_1_ receptor [21, 22]. However, a recent in vivo study showed that intravenous infusion of PAC_1_ receptor antibody, inhibited evoked nociceptive activity in the trigemino-cervical complex in rats, and these results were comparable to the inhibition observed with sumatriptan [123]. These results have led to the development of antibodies against PACAP (ALD1910) and PAC_1_ receptor (AMG 301) for migraine treatment.

In conclusion, the data presented in this review indicate that PACAP and PAC_1_ receptor blockade are promising migraine therapies but results from clinical trials are needed in order to confirm their efficacy and their side effects profile.

## Abbreviations

AC: Adenylyl cyclase
BBB: Blood-brain barrier
CGRP: Calcitonin gene-related peptide
CH: Cluster headache
CNS: Central nervous system
MCA: Middle cerebral artery
MMA: Middle meningeal artery
MRA: Magnetic resonance angiography
PACAP38: Pituitary adenylate cyclase activating polypeptide-38
PLC: Phospholipase C
PTSD: Post-traumatic stress disorder
TG: Trigeminal ganglion
TNC: Trigeminal nucleus caudalis
VIP: Vasoactive intestinal peptide
